# Identifying and Validating Alcohol Diagnostics for Injury-Related Trauma in South Africa: Protocol for a Mixed Methods Study

**DOI:** 10.2196/52949

**Published:** 2024-03-11

**Authors:** Petal Petersen Williams, Megan Prinsloo, Margaret M Peden, Ian Neethling, Shibe Mhlongo, Sithombo Maqungo, Charles Parry, Richard Matzopoulos

**Affiliations:** 1 Mental Health, Alcohol, Substance use and Tobacco Research Unit South African Medical Research Council Cape Town South Africa; 2 Institute for Life Course Health Research Department of Global Health Stellenbosch University Cape Town South Africa; 3 Department of Psychiatry and Mental Health University of Cape Town Cape Town South Africa; 4 Burden of Disease Research Unit South African Medical Research Council Cape Town South Africa; 5 Institute for Lifecourse Development Faculty of Education, Health & Human Sciences University of Greenwich London United Kingdom; 6 School of Public Health University of Cape Town Cape Town South Africa; 7 George Institute for Global Health UK Imperial College London London United Kingdom; 8 WHO Collaborating Centre on Injury Prevention and Trauma Care London United Kingdom; 9 Gender and Health Research Unit South African Medical Research Council Pretoria South Africa; 10 Biostatistics Unit South African Medical Research Council Pretoria South Africa; 11 Orthopaedic Trauma Service Groote Schuur Hospital University of Cape Town Cape Town South Africa; 12 Department of Psychiatry Stellenbosch University Cape Town South Africa

**Keywords:** alcohol, diagnostic, surveillance, injuries, violence, road traffic, Africa, South Africa, alcohol misuse, alcohol diagnostics, addiction, mixed methods, trauma, risk, injury, diagnostic tools, diagnostic tool, clinical management, injury, injury prevention, accident, accidents

## Abstract

**Background:**

The burden of alcohol use among patients with trauma and the relative injury risks is not routinely measured in South Africa. Given the prominent burden of alcohol on hospital trauma departments, South Africa needs practical, cost-effective, and accurate alcohol diagnostic tools for testing, surveillance, and clinical management of patients with trauma.

**Objective:**

This study aims to validate alcohol diagnostics for injury-related trauma and assess its use for improving national health practice and policy.

**Methods:**

The Alcohol Diagnostic Validation for Injury-Related Trauma study will use mixed methods across 3 work packages. Five web-based focus group discussions will be conducted with 6 to 8 key stakeholders, each across 4 areas of expertise (clinical, academic, policy, and operational) to determine the type of alcohol information that will be useful for different stakeholders in the injury prevention and health care sectors. We will then conduct a small pilot study followed by a validation study of alcohol diagnostic tools (clinical assessment, breath analysis, and fingerprick blood) against enzyme immunoassay blood concentration analysis in a tertiary hospital trauma setting with 1000 patients. Finally, selected alcohol diagnostic tools will be tested in a district hospital setting with a further 1000 patients alongside community-based participatory research on the use of the selected tools.

**Results:**

Pilot data are being collected, and the protocol will be modified based on the results.

**Conclusions:**

Through this project, we hope to identify and validate the most appropriate methods of diagnosing alcohol-related injury and violence in a clinical setting. The findings from this study are likely to be highly relevant and could influence our primary beneficiaries—policy makers and senior health clinicians—to adopt new practices and policies around alcohol testing in injured patients. The findings will be disseminated to relevant national and provincial government departments, policy experts, and clinicians. Additionally, we will engage in media advocacy and with our stakeholders, including community representatives, work through several nonprofit partners to reach civil society organizations and share findings. In addition, we will publish findings in scientific journals.

**International Registered Report Identifier (IRRID):**

DERR1-10.2196/52949

## Introduction

Alcohol indicators obtained from patients seen in emergency departments or admitted to hospitals are one of the key and most cost-effective data sources for estimating the impact of alcohol on communities and health [1] to quantify problem drinking, alcohol-impaired driving, trauma readmissions, and premature death [2]. This assists in identifying high-risk groups that should be targeted for prevention. Effective monitoring of alcohol-related morbidity and mortality requires the collection of alcohol-related indicators, in which regular reports on the key predefined indicators are submitted by hospitals, primary health care units, or emergency services [3,4].

The contribution of alcohol to the global burden of disease is undisputed. In addition to risks such as noncommunicable diseases, infectious diseases, and mental health problems as a result of hazardous and harmful alcohol use [3], the trauma burden of intentional and unintentional risk of injury from alcohol is a major public health concern. Studies from sub-Saharan Africa have highlighted the impact of alcohol on injury and violence [5-8] and concerns over alcohol consumption and alcohol-attributable burden of disease. The lack of attention alcohol-related harm receives from policy makers has been raised [9,10], with calls for stronger and more effective alcohol control measures.

In South Africa, approximately a third (31.2%) of alcohol-attributable deaths in 2012 occurred as a result of injuries, while 15.9% and 12.8% of alcohol-attributable disability-adjusted life years were caused by road traffic injuries and interpersonal violence, respectively [11]. The adult per capita consumption of alcohol in South Africa is extremely high (64.6 g of absolute alcohol per drinker per day). Almost 6 out of 10 South African drinkers older than the age of 15 years are reported to engage in heavy episodic drinking [3], which is strongly associated with increased injury risk.

COVID-19, and the related alcohol bans in South Africa, has brought the impact of alcohol on trauma presenting to health facilities into sharp focus in the country [12]. Moultrie et al [13] and Barron et al [14] demonstrated how a total ban on alcohol during the COVID-19 pandemic resulted in significantly fewer injury deaths. However, the absence of routine and reliable alcohol-related injury surveillance data have been identified as a critical gap, and the government has had to rely on the South African Medical Research Council’s (SAMRC) rapid mortality reporting [15] of all injury-related deaths and ad hoc surveillance studies [13,16] to demonstrate the association between the availability of alcohol and alcohol harm. This gap has substantially hindered the ability to provide regular information on changes in the pattern of alcohol-related injuries and limits the ability to measure the impact of policy changes, implementation, and enforcement.

As the country transitions from the COVID-19 crisis response, it is likely that there will be further pressure on the government—from civil society and health and social agencies—to implement more sustained intervention strategies to reduce harmful drinking and to monitor the impact of any interventions on the alcohol-related injury burden. This has highlighted the absence of practical, cost-effective, and accurate alcohol diagnostic tools in the South African trauma setting. Accurate measurement would improve surveillance and influence the clinical management of trauma, inform and improve government policies to address heavy drinking, and assess the impact of alcohol policy reform. The proposed study thus aims to determine the type of information that will be useful for stakeholders in the trauma care and injury prevention sectors, to validate the efficacy of a selection of alcohol diagnostic tools, and to explore their feasibility for wider provincial or national implementation as a routine source of information on the alcohol relatedness of injuries.

## Methods

### Study Design and Setting

**Figure 1:**
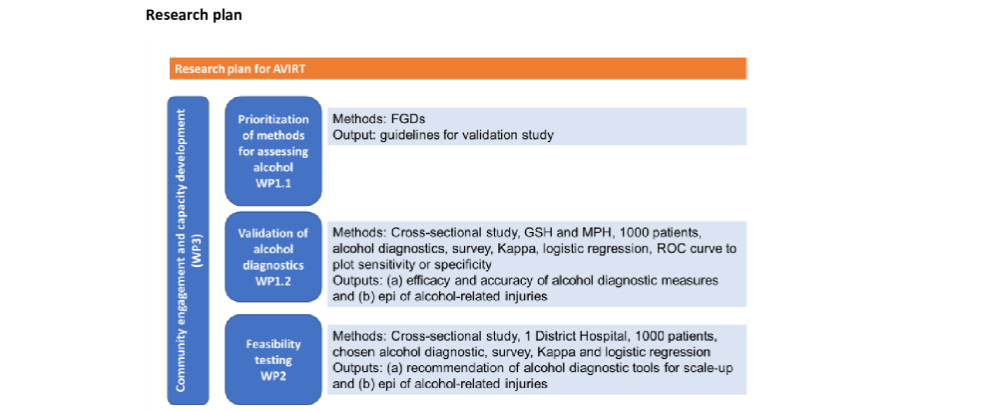
Diagrammatic illustration of the 3 studies and anticipated outputs. AVIRT: Alcohol Diagnostic Validation for Injury-Related Trauma; GSH: Groote Schuur Hospital; MPH: Mitchell’s Plain Hospital.

We will use a mixed methods participatory approach across 3 work packages (WPs) to validate alcohol diagnostics for injury-related trauma and assess its use for improving national health practice and policy (Figure 1). Outcomes from this research will inform health practice, policy development, and sustained intervention strategies. A validation study will be conducted in the trauma unit of 2 public health facilities in the Western Cape Province. Further testing will be conducted in 1 hospital setting in the Western Cape Province. These 2 hospitals represent high injury caseloads, particularly for violence and road traffic injuries [17,18].

### Research Plan

#### WP1: Prioritization and Validation of Methods for Assessing Alcohol Use

We will conduct focus group discussions (FGDs) with various stakeholders to ascertain the current alcohol assessment practice in hospitals and the type of information that could assist in the acute management of injured patients and measure alcohol use for public health surveillance. This will be followed by a cross-sectional study to validate the efficacy of three diagnostic tools: (1) clinical assessment, which is based on a clinician’s observation of apparent intoxication classifies the alcohol status of a patient into 4 categories as per International Classification of Diseases, Tenth Revision (ICD-10) coding (Y91.0 mild, Y91.1 moderate, Y91.2 severe, and Y91.3 very severe) [19]; (2) breathalyzers provide an estimation of the ethanol content in the breath [20]; and (3) fingerprick test to provide a capillary blood reading in 1-2 minutes and is used to facilitate the clinical process, as well as the use of the ICD-10 Y91 coding [19].

These 3 index tests will be measured against venous blood testing for the presence of ethanol as the reference standard, for which we will use enzyme immunoassay [21] for testing of blood alcohol concentration (BAC). The analysis will be conducted by a private pathology laboratory.

This WP will also describe the epidemiology of alcohol-related injuries among trauma cases presenting at the emergency unit of the selected trauma units through a prospective study. Patients presenting to the trauma units for first-time treatment of their injuries will be interviewed to record patient demographics, the injury intent, and related mechanisms and will be tested for alcohol, using the aforementioned alcohol diagnostic tools. Prior to the validation study, a small pilot study will be conducted over 2 weeks to test the consent procedures, the study questionnaire platform, and the logistics surrounding the blood withdrawal and use of a courier to the contracted laboratory for centrifugation within the 2-hour limit to preserve the alcohol.

#### WP2: Field Testing Selected Diagnostic Tools

Guided by findings from WP1, the alcohol diagnostic tools will be field tested in a district hospital in a large suburb on the outskirts of Cape Town. This WP aims to test the suitability of validated alcohol diagnostic methods for routine use in a hospital trauma setting on a day-to-day basis.

#### WP3: Community Engagement and Capacity Development

Engagement with clinicians, operational stakeholders (eg, nongovernmental organizations and emergency services), and individuals working in the policy arena are key to the success of any policy change. These stakeholders will be engaged through the FGDs at the commencement and again at the end to include community and patient representation. Three sets of activities for this phase will thus include:

- Step 1: FGDs on the use of the recommended methods and what would be required for implementation and the role of the measure in health care provision;
- Step 2: workshops or webinars with stakeholders for input on findings and to convey recommendations for uptake and integration; and
- Step 3: community engagement and recommendations for uptake and integration within the health system.

Additionally, capacity development through linking research to policy and practice for increased commitment and support by the government to fund and implement further scale-up of this study in trauma facilities nationally will be threaded through the project.

### Study Population and Sampling Procedure

The Applying Research to Policy and Practice for Health (ARCH) stakeholder mapping tool will be used to guide our mapping process [22] to identify stakeholders to participate in FGDs in WP1. FGDs will consist of approximately 6 to 8 participants each, and we anticipate holding 5 FGDs or until theoretical saturation is reached. We will start by making a list of stakeholders in the trauma or injury prevention and alcohol policy fields according to 4 categories: academic, clinical, operational, and policy stakeholders. Snowballing techniques from the initial core group will be used to identify additional stakeholders. Stakeholders will then be placed in a power-interest matrix based on the information outlined in the mapping [22] in order to categorize South African trauma and injury stakeholders according to their role in the professional landscape, to understand methods of engagement, and to lay out the proposed engagement strategy for the stakeholders throughout the project. FGDs will be exploratory and use a descriptive and contextual design [23]. Participants identified through this process will be invited to take part and required to provide informed consent before participation. FGDs with participants will take place over a 2-month period and will take place digitally to accommodate stakeholders in different locations. These FGDs will be guided by a semistructured interview sheet and run for between 45 and 60 minutes. They will be conducted by a trained facilitator in English.

For inclusion in the WP1 validation study, consenting patients presenting at the trauma units will be 18 years and older and injured <6 to 8 hours prior to arrival at the facility. All new consecutive admissions meeting the inclusion criteria will be tested for alcohol use. Unconscious, ventilated patients, and intoxicated patients with a breathalyzer (BrAC) test result >0.10 mg/L, who will be regarded as too intoxicated for informed consent, will require delayed consent to participate. Patients with severe cognitive impairment will be excluded. If the capacity to consent later is not regained, patients will be excluded. The required sample size to validate and assess the diagnostics’ performance is estimated to be 1000 patients. This is based on the eligibility criteria and a targeted sample of cases stratified into a 5-level category variable of intoxication and ICD-10 Y91 severity (Multimedia Appendix 1). From prior studies [24], we expect that 40% will be ineligible for study entry, and a further 60% of the 600 eligible participants will have no alcohol detected or below 0.05 g/100 mL as the legal driving limit. The remaining 4 groups with a positive alcohol detection (“a patient who has a BAC reading of 0.05 g/100 mL” or above) will be closely monitored to ascertain that a minimum of 60 cases per BAC versus ICD-10 Y91/diagnostic measurements code category is captured. Using the expected 60%/40% split in zero versus positive for alcohol, we expect 360 zero alcohol and 240 alcohol-related cases; we do, however, expect that the distribution will vary across BAC categories.

Based on these assumptions, and to test a strict margin that the null hypothesis (*k*_0_=0.7) and the alternative hypothesis (*k*_1_=0.8) will be considered as substantial agreement, further input variables to determining the sample size included the 5 categories with frequencies equal to 0.6 (proportion of 0 alcohol cases) and 0.1 (for each proportion of the 4 remaining alcohol-positive categories). Based on this, we are expecting a sample size of 396 participants at 90% power. This power calculation is based on a significance level of *P*<.05. Hence, we expect our targeted sample of 600 eligible cases to be enough.

The same sampling strategy will be applied to select patients for the WP2 field testing of validated alcohol diagnostic tools in a district hospital trauma setting and estimated to be approximately 1000 patients. For WP2, the alcohol diagnostic tools will be used in separate data collection periods for ease of use. As the selected district hospital has a monthly average caseload of approximately 561 cases [18], we will plan to collect an approximate sample of 400 eligible cases for 1 alcohol diagnostic tool (breathalyzer), followed by a week’s break, then for an additional 400 eligible cases to test a second diagnostic tool (fingerprick test) over the study’s planned duration of 90 days.

For WP3, hospital staff from the validation of alcohol diagnostics and field testing will be invited to participate in FGDs (step 1). These will follow the same procedures described previously for the prioritization of methods, and we will include a summary of relevant updated study information. In step 2, national or provincial policy and health management stakeholders and the hospital staff from step 1 will be invited to participate in a feedback workshop to lobby for uptake and implementation nationally and provincially. They will be complemented by approximately 30 key public health, clinician, health management, injury prevention, and policy stakeholders identified using the ARCH stakeholder mapping tool. Step 2 will be held in a hybrid web-based and in-person workshop at the SAMRC in Cape Town after the FGD data have been analyzed. For step 3, we will work with the SAMRC Corporate and Marketing Communications division for policy brief, infographic, and short video design or production. This is for dissemination and engagement with community stakeholders, for research translation and recommendations to national or provincial policy and health management stakeholders, suggesting uptake and integration to other hospital trauma facilities.

### Data Collection

The FGDs in WP1 will be conducted to explore stakeholders’ knowledge and views on current alcohol indicators collected in trauma settings and gaps in the collection of indicators. We will also obtain opinions from these stakeholders about diagnostic tools, implementation barriers, facilitators, feasibility, acceptability and the appropriateness of collecting routine, and reliable injury surveillance and alcohol-related harm. The findings from this substudy will inform the study on the validation of alcohol diagnostic measures.

For the validation of alcohol diagnostics (WP1), a draft survey questionnaire was adapted from the WHO Collaborative Study on Injuries and Alcohol, a study validated across multicountry sites globally [24]. Information on the injury intent, mechanism, clinical screening according to ICD-10 code Y91, and breathalyzer analysis were retained with additions including the 2 alcohol diagnostic tools (the withdrawal of blood for testing and the fingerprick measure), time of blood withdrawal and fingerprick, the South African Triage Scale, an indication of delayed consent, referring hospital name (if applicable), and hospital folder number.

Fieldworker study nurses will be employed and trained on informed consent and completion of the survey content, and the questionnaire or data capturing on the Kobotools platform [25], using the study’s electronic tablet devices. Fieldwork will occur over a 3-month period, with regular monitoring of the caseload to inform any adjustments to the schedule until the targeted sample of 1000 cases is achieved, of which 600 cases are estimated to be eligible. Study nurses will be required to work midweek during daytime hours as required and during night duty hours of 7 PM to 7 AM over weekends.

Blood samples will be couriered on a regular basis to a Pathcare laboratory near Groote Schuur Hospital. Preservation of the alcohol will be done by centrifuging the samples within 2 hours of blood withdrawal to separate the plasma from the serum and to have it sealed in a separately labeled tube for courier and analysis at a second Pathcare facility identified for blood alcohol testing.

For WP2, the survey questionnaire will be revised for the field testing phase of validated alcohol diagnostics with the addition of questions on the body region injured (head, face, neck, thorax, etc), the nature of injury (fracture, cut, bruise, concussion, etc), the selected alcohol diagnostic tools (Table 1), the patient’s drinking history prior to injury, a self-assessment on alcohol intoxication, and information on socioeconomic status.

**Table 1:** Summary of alcohol diagnostic screening tool measures.

Alcohol diagnostics	Data source
Clinical assessment: An observational assessment of alcohol intoxication conducted by a trained nurse or medical doctor. The assessment measures the severity of speech impairment, motor coordination, behavioral disturbances, etc through the use of a Likert scale (none to very severe) using ICD-10^a^ Y91 codes	WHO^b^ evidence of alcohol involvement is determined by the level of intoxication (ICD-10 Y91 codes) [26]
Active breathalyzer testing: The blood alcohol concentration measured in breath alcohol (BrAC) mg/L in exhaled breath through a straw. The minimum breath alcohol content detected is 0.03 mg/L BrAC. Alcohol can be detected up to 6-12 hours after the last drink. This method will not be used on ventilated patients Passive breathalyzer testing: Exhaled breath for all patients to indicate the presence or absence of breath alcohol (yes or no)	Dräger: SANAS-accredited breathalyzer screener used by blowing through a sterile mouthpiece for a digital reading (active) Passive: indicate the presence or absence of breath alcohol (yes or no)
Fingerprick test: Accurate measurement of capillary whole blood samples collected via fingerprick using a lancet. Results displayed in %BAC, mg/L, or μg/dL BrAC. The lowest level of detection is 0.03% BAC	Fingerprick test: rapid blood alcohol meter
Blood sample: Detecting the presence or absence of ethanol in the blood [27] using enzyme immunoassay. Venous blood was collected by qualified nurses in a sodium fluoride tube; alcohol levels were reported in g/dL	Testing with a venous blood sample

^a^ICD-10: International Classification of Diseases, Tenth Revision.

^b^WHO: World Health Organization.

Fieldwork will be based on an idealized week, with a selection of hours across day- and nighttime, during the 3-month period. This is to ensure that the data are representative in terms of the facilities’ operating conditions, which are expected to change by time of the month, hour, weekday or weekend, and so forth to assess the suitability of the alcohol diagnostic tools for possible national scale-up.

In WP3, FGDs with participants in step 1 will take place digitally to accommodate stakeholders in different locations. Interview guides will consist of open-ended questions to explore participants’ views on the recommended methods, their thoughts on the requirements for implementation (provincially and nationally), and what the role of the measure would be in health care provision more broadly. Specific areas to discuss will include (1) intervention content; (2) intervention delivery; (3) strategies for addressing possible barriers to intervention delivery; (4) research questions around acceptability, feasibility, and sustainability of measuring alcohol-related trauma; and (5) strategies to generate local stakeholder buy-in. These areas of discussion will ensure appropriate information are collected that will inform national stakeholders on the benefits for the adoption of the recommended alcohol diagnostic screening tools for larger implementation [28].

For step 2, the findings from WP1 and WP2, as well as the FGD results from WP3 (step 1) will be shared in a workshop with researchers; clinicians and hospital managers; traffic officials; emergency medical services; police, pathology, and rescue services; and national and provincial policy makers. The output will be a framework to guide the implementation and scale-up of routine diagnostic measuring of alcohol-related trauma. Workshop participants will be asked to evaluate the usefulness of the workshop and their satisfaction with the outcome.

### Data Analysis

WP1 and WP3 FGDs will be audio recorded and transcribed verbatim. Thematic analysis will be conducted based on deductive themes focusing on the exploration of current practices, implementation barriers, facilitators, feasibility, acceptability, and appropriateness of conducting alcohol diagnostics in public health facilities (WP1.1) and on acceptability, feasibility, and appropriateness of the recommended measure (WP3). The data will be managed using qualitative data analysis software NVivo (version 12; Lumivero) and will be presented in line with COREQ (Consolidated Criteria for Reporting Qualitative Research) guidance for reporting qualitative research [29].

For the validation of alcohol diagnostics in WP1 and WP2, data collection will be regularly monitored and managed by the study statistician. The blood alcohol analysis results will be merged into the main database using the unique case ID. The correlation coefficients of the continuous alcohol diagnostic measurements and the blood alcohol tests will be reported and represented graphically. A Kappa statistic [30] will be used to assess the level of agreement between BAC categories and the various alcohol diagnostic tests and the clinical assessment as per ICD-10 (International Classification of Diseases) Y91 codes. Taking into consideration that the alcohol diagnostic measures are ordinal responses, we will use ordinal regression to model the receiver operating characteristic (ROC) curve [31,32]. This diagnostic test plots sensitivity against the specificity of the alcohol diagnostics measures against the gold standard BAC. The area under the ROC curve will also be used to characterize the accuracy of the diagnostic tests, providing all information on its performance, instead of only a single estimate of the test’s sensitivity and specificity. The trade-offs between the ROC curve’s sensitivity and specificity can then be assessed to inform a decision threshold [33,34] for the alcohol diagnostic methods to be used in phase 2. Any level above 70%, sensitivity and specificity will be acceptable and is usually considered as “fair,” followed by 80% as “good” and 90% as “excellent.”

If multiple tests meet the same criteria, we will use the area under the curve to determine the best test. We will, however, proceed to test the feasibility of use for both the breathalyzer and the Fingerprick test if they meet the specified sensitivity and specificity criteria. Besides reporting the results of validating the alcohol diagnostic tools, descriptive statistics for analysis will include age and gender, followed by an analysis of the severity of the injury (assessed by the triage scale), the intent of the injury (violence, road traffic, unintentional, and self-harm), the related mechanism of injury (ie, gunshot, stabbing, pedestrian, driver, fall, etc), and BAC categories. Data analysis will be conducted on STATA (version 17; Stata Corp). Data will be presented in line with STROBE (Strengthening the Reporting of Observational Studies in Epidemiology) guidance for reporting cross-sectional studies [35].

For the field testing (WP2), we will follow the same data management and analysis procedures and packages as for the validation of alcohol diagnostics. Further analysis will include patient demographics, the nature of injury (fracture, sprain, open wound, burn, etc) and body region injured, the intent of the injury (violence, road traffic, unintentional, and self-harm) and the related mechanism of injury (ie, gunshot, stabbing, pedestrian, driver, fall, etc), and triage scale. We will use ordinal regression to analyze the severity of injuries (as indicated by the triage scale) as the outcome, and the level of intoxication (indicated as 0.05 g/100 mL and above) as one of the independent variables. In addition, a multinomial regression will be used to assess the association between the outcome as the type of injury with the level of alcohol intoxication. As information on drinking prior to injury will be recorded to determine the type of alcohol and volume consumed, the dose-response relation between the number of drinks consumed within 6 hours leading up to the injury and the relative risk of being injured will be analyzed and reported. Socioeconomic information on employment status, household income, and suburb of residence will be captured and used to inform alcohol policies.

For the workshop evaluation form in WP3, we will sum each evaluation item to create scores for the evaluation form and will summarize using mean with the SD or median with IQR.

### Patient and Public Involvement

Clinicians are an important participant group and were involved in the initial design of the study and as coinvestigators. We will be using a participatory approach through stakeholder engagement initiated in the prioritization of methods to include community and patient representation; then incorporating a synthesis of findings from the validation of alcohol diagnostics and suitability testing to conduct qualitative research on the use of the recommended alcohol diagnostic method. Feedback will be sought on requirements for uptake and integration within the health system, which has a specific focus on community-based participatory research for study synthesis and recommendations. This final uptake activity is iterative and dependent on the outcomes of the formative work. Therefore, the study overall is geared to soliciting participant co-design.

### Ethical Considerations

Ethical approval for the study has been granted by the research ethics committee of the South African Medical Research Council (EC005-2/2022) and approval from the Western Cape Health Department. Written informed consent will be obtained from all study participants, and all data will be anonymized. No compensation will be provided for study participation.

## Results

Pilot data are being collected, and the protocol will be modified based on the results. The findings will be disseminated to relevant national and provincial government departments, policy experts, and clinicians. We will publish findings in scientific journals, engage in media advocacy, and share findings with our stakeholders, including community representatives, nonprofit partners, and civil society organizations.

## Discussion

There is a lack of routine and reliable injury surveillance specifically alcohol-related harm data in South Africa to respond to the related trauma burden. Additionally, research to develop systems or reliable mechanisms to test alcohol-related trauma and to monitor the impact of interventions is lacking. Alcohol is an established risk factor for violence and injuries and accurately monitoring alcohol relatedness in response to interventions, policy changes, and so forth will be necessary to evaluate effectiveness as suggested by the WHO SAFER strategy of “*Monitoring*,” 1 of 3 essential strategies aimed at government officials for the purpose of developing evidence-based alcohol policies and action plans to address alcohol harm [36]. The prominent role of alcohol in the trauma setting has become particularly pronounced during the COVID-19 lockdown, and this study will provide crucial evidence needed for the effective measurement of alcohol-related trauma to improve injury surveillance and clinical management. A few limitations of the study have been identified. First, expert stakeholders included in FGDs will be restricted to known contacts within the field. However, by using the ARCH stakeholder mapping tool to identify appropriate stakeholders, we are confident that we will involve relevant individuals, groups, organizations, and institutions and engage with them in a manner that can contribute to successful research uptake [22]. Second, the validity testing of the alcohol diagnostics will use enzyme immunoassay instead of the gold standard gas chromatography method. The detection of ethanol by gas chromatography, which has the advantage of being able to separate ethanol from other alcohol, is widely considered as the “gold standard” for alcohol measurement [21,27]. Due to the cost implications for this method of testing and the fact that we will not be using the results of this study in medicolegal cases, we will be using enzyme immunoassay [21] for testing of blood alcohol and will consider this as the gold standard for the purpose of this study. Third, validity testing at a tertiary hospital could influence the cut-off period to detect alcohol within the blood due to referrals from primary and secondary health facilities. However, the selected tertiary hospital was specifically selected as the site for validating the alcohol diagnostic tools, as it is a large tertiary hospital situated in Cape Town, South Africa, where the trauma unit is burdened by high injury caseloads, particularly for violence and road traffic injuries [16,17,37]. Through this study, we hope to identify and validate the most appropriate methods of diagnosing alcohol-related injury and violence in clinical settings. The findings from this study are likely to be highly relevant and could influence our primary beneficiaries—policy makers and senior health clinicians—to adopt new practices and policies around alcohol testing in injured patients.

## Appendix

Multimedia Appendix 1 Targeted sampling strategy with minimum sample requirement, prior to eligibility assumptions by blood alcohol concentration reading and Y91 code.

## Abbreviations

ARCH: Applying Research to Policy and Practice for Health
BAC: blood alcohol concentration
COREQ: Consolidated Criteria for Reporting Qualitative Research
FGD: focus group discussion
ICD-10: International Classification of Diseases, Tenth Revision
ROC: receiver operating characteristic
SAMRC: South African Medical Research Council
STROBE: Strengthening the Reporting of Observational Studies in Epidemiology
WP: work package

## Data Availability

The data generated and analyzed during this study was subject to restricted consent from participants. The data can be made available by the corresponding author upon reasonable request.

## References

[1] Flynn A, Wells S (2013). Community indicators: assessing the impact of alcohol use on communities. Alcohol Res.

[2] Rootman DB, Mustard R, Kalia V, Ahmed N (2007). Perceptions and realities of testing for alcohol and other drugs in trauma patients. J Trauma.

[3] (2018). Global Status Report on Alcohol and Health 2018. Geneva: World Health Organization.

[4] Parry CDH, Trangenstein PJ, Erasmus J, Diedericks A, Harker N (2020). Developing indicators to measure the implementation of the Western Cape alcohol harms reduction strategy in South Africa. Afr J Drug Alcohol Stud.

[5] Staton CA, Vissoci JRN, Toomey N, Abdelgadir J, Chou P, Haglund M, Mmbaga BT, Mvungi M, Swahn M (2018). The impact of alcohol among injury patients in Moshi, Tanzania: a nested case-crossover study. BMC Public Health.

[6] Liebenberg J, du Toit-Prinsloo L, Saayman G, Steenkamp V (2021). Prevalence and profile of drugs and alcohol in fatally injured drivers in Pretoria, South Africa. Afr Saf Promot.

[7] Greene MC, Kane JC, Tol WA (2017). Alcohol use and intimate partner violence among women and their partners in sub-Saharan Africa. Glob Ment Health (Camb).

[8] Meel BL (2006). Alcohol-related traumatic deaths in Transki region, South Africa. Internet J Medical Update.

[9] Ferreira-Borges C, Parry CDH, Babor TF (2017). Harmful use of alcohol: a shadow over sub-Saharan Africa in need of workable solutions. Int J Environ Res Public Health.

[10] Morojele NK, Dumbili EW, Obot IS, Parry CDH (2021). Alcohol consumption, harms and policy developments in sub-Saharan Africa: the case for stronger national and regional responses. Drug Alcohol Rev.

[11] Matzopoulos R, Cois A, Probst C, Parry CDH, Vellios N, Sorsdahl K, Joubert JD, van Wyk VP, Bradshaw D, Pacella R (2022). Estimating the changing burden of disease attributable to alcohol use in South Africa for 2000, 2006 and 2012. S Afr Med J.

[12] Matzopoulos R, Walls H, Cook S, London L (2020). South Africa's COVID-19 alcohol sales ban: the potential for better policy-making. Int J Health Policy Manag.

[13] Moultrie TA, Dorrington RE, Laubscher R, Groenewald P, Parry CDH, Matzopoulos R, Bradshaw D (2021). Unnatural deaths, alcohol bans and curfews: evidence from a quasi-natural experiment during COVID-19. S Afr Med J.

[14] Barron K, Parry CDH, Bradshaw D, Dorrington R, Groenewald P, Laubscher R, Matzopoulos R (2022). Alcohol, violence and injury-induced mortality evidence from a modern-day prohibition. Rev Econ Stat.

[15] Bradshaw D, Laubscher R, Dorrington R, Groenewald P, Moultrie T (2021). Report on weekly deaths in South Africa 1 January—29 December 2020 (week 52). South African Medical Research Council.

[16] Navsaria PH, Nicol AJ, Parry CDH, Matzopoulos R, Maqungo S, Gaudin R (2020). The effect of lockdown on intentional and nonintentional injury during the COVID-19 pandemic in Cape Town, South Africa: a preliminary report. S Afr Med J.

[17] Nicol AJ, Sorsdahl K, Hoffman R (2013). Violence and substance use at a Cape Town trauma centre.

[18] Parak M (2021). Hospital and Emergency Centre Tracking Information System (HECTIS) trauma data. Western Cape Government.

[19] Touquet R, Harris D (2012). Alcohol misuse Y91 coding in ICD-11: rational terminology and logical coding specifically to encourage early identification and advice. Alcohol Alcohol.

[20] Hadland SE, Levy S (2016). Objective testing: urine and other drug tests. Child Adolesc Psychiatr Clin N Am.

[21] Horvat V, Mandic S, Mandic D, Nestic M, Jonjic J, Majetic-Cetina N (2018). Comparison of enzyme immunoassay and gas chromatography method for determination of whole blood and urine alcohol levels.

[22] Mtika W, Wicox H, De Colombi NF (2021). Stakeholder mapping tool for applying research to policy and practice for health (ARCH). The Global Health Network.

[23] Moriarty J (2011). Qualitative methods overview. School for Social Care Research.

[24] (2007). WHO collaborative study on alcohol and injuries: final report. World Health Organization.

[25] (2018). Simple, robust and powerful tools for data collection. Kobotoolbox.

[26] (2019). ICD-10 Y91 codes: evidence of alcohol involvement determined by level of intoxication. World Health Organization.

[27] Jones AW (2019). Alcohol, its analysis in blood and breath for forensic purposes, impairment effects, and acute toxicity. WIREs Forensic Sci.

[28] Scott SD, Plotnikoff RC, Karunamuni N, Bize R, Rodgers W (2008). Factors influencing the adoption of an innovation: an examination of the uptake of the Canadian Heart Health Kit (HHK). Implementation Sci.

[29] Tong A, Sainsbury P, Craig J (2007). Consolidated criteria for reporting qualitative research (COREQ): a 32-item checklist for interviews and focus groups. Int J Qual Health Care.

[30] Cohen J (1968). Weighted kappa: nominal scale agreement with provision for scaled disagreement or partial credit. Psychol Bull.

[31] Hanley JA, McNeil BJ (1982). The meaning and use of the area under a Receiver Operating Characteristic (ROC) curve. Radiology.

[32] Hanley JA, McNeil BJ (1983). A method of comparing the areas under receiver operating characteristic curves derived from the same cases. Radiology.

[33] Tosteson AN, Weinstein MC, Wittenberg J, Begg CB (1994). ROC curve regression analysis: the use of ordinal regression models for diagnostic test assessment. Environ Health Perspect.

[34] Luo J, Xiong C (2012). DiagTest3Grp: an R package for analyzing diagnostic tests with three ordinal groups. J Stat Softw.

[35] Cuschieri S (2019). The STROBE guidelines. Saudi J Anaesth.

[36] (2019). The SAFER Technical Package: Five Areas of Intervention at National and Subnational Levels.

[37] Nicol A, Knowlton LM, Schuurman N, Matzopoulos R, Zargaran E, Cinnamon J, Fawcett V, Taulu T, Hameed SM (2014). Trauma surveillance in Cape Town, South Africa: an analysis of 9236 consecutive trauma center admissions. JAMA Surg.

