# Supplementary material for: Identifying and Validating Alcohol Diagnostics for Injury-Related Trauma in South Africa: Protocol for a Mixed Methods Study
Source: JMIR Res Protoc. 2024 Mar 11;13:e52949. doi: 10.2196/52949 (PMC10964142; doi:10.2196/52949)
Supplement: Multimedia Appendix 1 [file resprot_v13i1e52949_app1.pdf]

Table S1.

Targeted sampling strategy with minimum sample requirement, prior to eligibility assumptions by BAC reading and Y91 code

	**BAC reading (g/100 mL[a]) by severity of alcohol intoxication**					
	None[b] (0.000-0.049)	Mild (0.050-0.099)	Moderate (0.100-0.199)	Severe (0.200-0.299)	Very severe (0.300 +)	Min. Total
Zero alcohol	360					360
Y91.0 Mild alcohol intoxication		60				60
Y91.1 Moderate alcohol intoxication			60			60
Y91.2 Severe alcohol intoxication				60		60
Y91.3 Very severe alcohol intoxication					60	60
Min. Total						600

[a]BAC readings for BrAC collected by the Alcostick and Breathalyzer will be converted to g/100 mL during the analysis stage.

[b] A person is assumed to be under the influence of alcohol when the quantity consumed exceeds the alcohol tolerance of an individual, and causes impairment in mental and physical ability. These effects are usually influenced by several factors such as metabolism, consumption history, body fat content, and pre-existing medical conditions (Greeley, J., & McDonald, D. (1992). Research Paper 14: Alcohol and Human Behaviour. In D. Biles & D. McDonald (Eds.), *Deaths in custody Australia 1980-89*. Canberra: Australian Institute of Criminology.). Hence, the corresponding scale is used for Table S1 in Multimedia Appendix 1 and BAC levels within the legal South African driving limit of <0.05g/100 mL will be excluded for the analysis of "alcohol intoxication."
